# Dermatochirurgisch optimierte Ohrtamponade – eine Technik zum sicheren Verschluss des Meatus acusticus externus

**DOI:** 10.1111/ddg.70025

**Published:** 2026-10-05

**Authors:** Martin Flatz, Sandra Schreiner, Christian Kunte

**Affiliations:** ^1^ Artemed Fachklinik München, Mozartstraße 14a‐16 München Germany

## EINLEITUNG

Im Rahmen (dermato‐)chirurgischer Eingriffe am Kopf, vor allem temporal, präaurikulär sowie am Ohr selbst, besteht stets die Gefahr der Verlegung des Meatus acusticus externus durch intraoperative Blutungen mit Bildung okkludierender Blutkoagel. Ursächlich kann unter anderem eine Verletzung des Ramus auricularis anterior der Arteria (A.) temporalis superficialis an der Vorderseite der Ohrmuschel sein. Insbesondere aber die gute Durchblutung des Tragus und des Meatus acusticus externus durch die A. auricularis profunda sowie der Ohrhelix, hier durch die *helical rim arcade*, kann im Rahmen operativer Eingriffe bei Blutungsereignissen zu einer Verlegung des Meatus acusticus externus führen.^1^ Während oberflächliche Fremdkörper in der Regel mittels einer Alligatorzange entfernt werden können,^2^ kann bei tiefsitzenden Blutkoageln postoperativ eine Intervention durch einen Hals‐, Nasen‐ und Ohrenarzt erforderlich sein.

Eine gängige Methode zum Schutz des Meatus acusticus externus ist die Einlage fetthaltiger Gazen, in unserem Fall einer paraffingetränkten Jelonet^®^‐Salbenkompresse (5 × 5 cm; Smith & Nephew, Hull, England). Diese können, neben der erschwerten Handhabung, das umliegende Gebiet stark einfetten und bergen das Risiko, während der Operation zu dislozieren. So stellt eine Verwendung dieser Gazen einen Kompromiss aus einer Sichteinschränkung auf den OP‐Situs bei zu oberflächlicher und aufwändiger Entfernung bei zu tiefer Positionierung dar. Alternativ können sterile Polyvinylalkohol‐(PVA)‐Ohrtamponaden (zum Beispiel Pope Ear Wick, 9 × 15 mm; Merocel^®^, Medtronic Xomed, Jacksonville, FL, USA), auch in Kombination mit einer fetthaltigen Gaze, verwendet werden. Im Folgenden wird eine weitere Option mit gängigen Materialien eines Operationssiebs für den intraoperativen Schutz des Meatus acusticus externus aufgezeigt.

## DURCHFÜHRUNG

Die folgende Technik beruht auf klinischer Erfahrung und wurde bereits in zahlreichen Fällen angewendet. Erst wird ein kleiner, steriler Präpariertupfer mit einer Nadel und einem Faden mittig durchstochen (Abbildung 1a). Im Rahmen der Präparation des Rückhohlbändchens wird der Faden durch den sterilen Präpariertupfer gezogen, bis das Fadenende noch circa 7–10 cm aus ihm herausragt. Die beiden Fadenenden werden parallel positioniert und gemeinsam mit einer Klemme fixiert (Abbildung 1b), der überschüssige Faden hinter der Klemme gekürzt. Abschließend wird der Präpariertupfer mit Hilfe der für den Verband bereitliegenden Gaze eingefettet. Hierfür kann die Abdeckung mit Gaze auf einer Seite angehoben und der Tupfer an der unteren Schutzschicht entlanggeführt werden (Abbildung 1c).

**ABBILDUNG 1:**
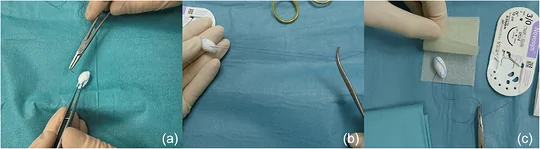
(a) Durchstechung des Präpariertupfers; (b) Präparation des Rückholbändchens; (c) Einfetten der Ohrtamponade mit einer Jelonet^®^‐Salbenkompresse zum erleichterten Einführen und zur Abdichtung gegenüber Feuchtigkeit.

In diesem Fall wird ein geflochtener 3/0 Faden mit einer DS24 Nadel verwendet und damit ein Präpariertupfer Gr. 2 (gebrauchsfertige Präpariertupfer aus Verbandmull, EN 14079) durchstochen. Eingefettet wird die Tamponade hier mittels einer Jelonet^®^‐Salbenkompresse.

Nun kann die Ohrtamponade in den Meatus acusticus externus eingeführt werden (Abbildung 2b). Dies wird durch den aufgetragenen Fettfilm erleichtert. Zudem schützt dieser den Meatus acusticus externus durch seine wasserabweisenden Eigenschaften vor dem Eindringen von Desinfektionsmittel und Blut (Abbildung 2a). Bei einer Vielzahl von Desinfektionsmitteln, wie beispielsweise Iod, Chlorhexidin oder höherprozentigen Alkoholen, besteht die Gefahr einer möglichen Ototoxizität – insbesondere bei Vorliegen eines perforierten Trommelfells.^3^ Dank des Rückholbändchens kann die Positionierung der Tamponade ausreichend tief im Meatus acusticus externus erfolgen, um eine gute Sicht zu erzielen und eine rasche postoperative Entfernung der Tamponade zu gewährleisten. Das Rückholbändchen kann außerhalb des OP‐Gebietes mittels einer Klemme an den sterilen Abdeck‐Tüchern fixiert werden (Abbildung 2b, Kreis).

**ABBILDUNG 2:**
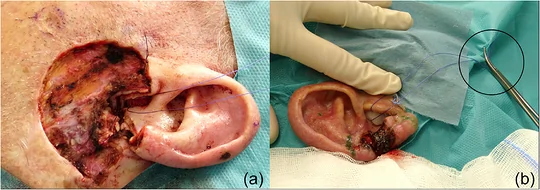
(a, b) Platzierung der Ohrtamponade; (b, Kreis) Fixierung des Rückholbändchens an der Abdeckung.

## ZUSAMMENFASSUNG

Die Verwendung beispielsweise einer Jelonet^®^‐Salbenkompresse ist für einfachere präaurikuläre Eingriffe gut geeignet. Bei komplexeren Eingriffen am Ohr mit erhöhtem Blutungsrisiko (Abbildung 2b) oder in unmittelbarer Nähe zum Meatus acusticus externus (Abbildung 2a) lässt sich die Ohrtamponade in wenigen Schritten vorbereiten und ist einer Paraffingaze in vielen Aspekten überlegen. Neben der erleichterten Applikation im Meatus acusticus externus und dessen zuverlässigen Verschluss ist die schnelle und sichere postoperative Entfernung der Tamponade ein großer Vorteil. Durch die hydrophoben Eigenschaften des zuvor eingefetteten Präpariertupfers und seiner Fähigkeit zur Absorption schützt er zuverlässig vor dem Eindringen von Flüssigkeit. Insbesondere die gute Übersicht über den OP‐Situs bei komplexen Eingriffen rechtfertigt in diesen Fällen den erhöhten zeitlichen Vorbereitungsaufwand (Abbildung 2a). Der Nachteil dieser Technik stellt der zeitliche Mehraufwand gegenüber der Applikation einer einfachen Salbenkompresse mit Paraffin dar. Dasselbe gilt im Vergleich zu der Verwendung einer Polyvinylalkohol (PVA)‐Ohrtamponade. Der Vorteil der beschriebenen Tamponade sind hier die verwendeten Materialien. Diese stehen meist als Standard in Eingriffs‐ und Operationsräumen zur Verfügung, während die Anschaffung einer PVA‐Ohrtamponade für den seltenen Bedarfsfall einen zusätzlichen Kostenfaktor darstellt.

## INTERESSENKONFLIKT

Keiner.
